# Clinical outcomes of over-the-top subscapularis repair in reverse shoulder arthroplasty

**DOI:** 10.1016/j.jseint.2024.02.010

**Published:** 2024-02-28

**Authors:** Robert J. Cueto, Kevin A. Hao, Daniel S. O’Keefe, Marlee A. Mallat, Keegan M. Hones, Lacie M. Turnbull, Jonathan O. Wright, Jose Soberon, Bradley S. Schoch, Joseph J. King

**Affiliations:** a College of Medicine, University of Florida, Gainesville, FL, USA; b Department of Orthopaedic Surgery and Sports Medicine, University of Florida, Gainesville, FL, USA; c Department of Orthopaedic Surgery, North Florida South Georgia Veterans Health System, Gainesville, FL, USA; d Department of Anesthesiology, North Florida Southern Georgia Veterans Health System, Gainesville, FL, USA; e Department of Anesthesiology, University of Florida, Gainesville, FL, USA; f Department of Orthopaedic Surgery, Mayo Clinic, Jacksonville, FL, USA

**Keywords:** Shoulder, Arthroplasty, Primary, Anatomic, Outcomes, Strength, Subscapularis management

## Abstract

**Background:**

Biomechanical research demonstrates increased subscapularis abduction range of motion (ROM) when the tendon’s upper two-thirds is repaired over-the-top of the center of rotation during reverse shoulder arthroplasty (RSA). This study compares the clinical outcomes of patients undergoing RSA with over-the-top subscapularis repair (OTTR) to patients without repair.

**Methods:**

We retrospectively reviewed 97 consecutive RSAs with either OTTR of the subscapularis (N = 75) or no repair (N = 22). Repair was attempted in all patients but not performed if the subscapularis could not be brought to the over-the-top position in 20° of external rotation (ER) and 30° of abduction. Improvements in ROM were compared to the minimal clinically important difference for RSA.

**Results:**

The mean follow-up was 3.8 ± 1.6 years. Demographics were similar between groups. Preoperatively, patients undergoing repair had greater ER when compared to those without repair (15 ± 16° vs. 5 ± 12°, *P* = .003). Postoperatively, patients undergoing repair had greater forward elevation (132 ± 21° vs. 126 ± 22°, *P* = .268) and abduction (114 ± 26° vs. 106 ± 23°, *P* = .193) with both exceeding the minimal clinically important difference (−2.9° and −1.9°, respectively); however, not statistically significant. Patients with repair were more frequently able to reach the small of their back postoperatively (65% vs. 21%, *P* = .006) but had less improvement in ER (13 ± 20° vs. 24 ± 20°, *P* = .028). Postoperative outcome scores, complications, and reoperations were similar between groups.

**Discussion:**

OTTR of the subscapularis in RSA had similar ROM and outcome scores compared to no repair, but a significantly larger proportion of patients with repair achieved functional internal rotation to the small of the back. ER limitations seen after conventional repair may also apply to this novel technique, but without a corresponding detrimental effect on forward elevation or abduction.

While there is general agreement about the benefit of reverse shoulder arthroplasty (RSA) to treat varying shoulder pathologies, how to manage the subscapularis muscle remains controversial.^16^^,^^19^ Those in support of repairing the subscapularis argue the potential for improved glenohumeral joint stability,^11^^,^^29^ improved internal rotation (IR) range of motion (ROM),^12^^,^^16^ and increased functional outcomes.^8^^,^^12^ Opponents to subscapularis repair point to the unfavorable biomechanics that arise due to its function as an internal rotator and an adductor with distalization of the humerus, with it acting as an antagonist to abduction, external rotation (ER), and forward elevation (FE), and question its roll in stability with modern implants.^7^^,^^8^ While thoroughly studied, data to guide clinicians regarding the most appropriate management of the subscapularis is often contradictory and without consensus.^6^^,^^10^^,^^16^^,^^19^^,^^27^^,^^30^^,^^37^^,^^40^ In a recent systematic review and meta-analysis of articles evaluating outcomes associated with management of the subscapularis tendon, De Fine and colleagues found that the subscapularis was repaired in 45% of included patients that underwent RSA, further highlighting the uncertainty from shoulder surgeons regarding whether or not to repair the subscapularis tendon.^10^

In this study, we present the clinical results of a novel technique for repairing the subscapularis, the over-the-top subscapularis repair (OTTR). In the OTTR, the superior two-thirds of the subscapularis tendon is repaired above the glenosphere center of rotation to the anterior aspect of the greater tuberosity. Previous biomechanical research by King et al^21^ demonstrated that OTTR increased the abduction moment arm and decreased the degree for which the subscapularis became an adductor when compared to standard repair. The OTTR of the subscapularis combines the purported benefits of subscapularis repair by maintaining the function of the subscapularis as an internal rotator while converting it from an adductor to an abductor through much of the glenohumeral arc of motion.^21^ While the biomechanics of the OTTR are promising, clinical studies reporting outcomes of this approach are needed.

The purpose of this study was to report clinical outcomes of patients who had undergone RSA with OTTR of the subscapularis. Secondarily, we compared patients who underwent OTTR with those who had no subscapularis repair. We hypothesized that patients who had undergone RSA with OTTR would have excellent clinical outcomes with similar forward flexion and abduction but improved IR compared to the no repair group.

## Materials and methods

After institutional review board approval, we retrospectively reviewed all RSAs performed from 2015 to 2019 by a single surgeon (J.J.K.) at one Veterans Health Administration hospital, representing a subset of his clinical practice. RSAs were grouped according to concomitant OTTR or subscapularis tenotomy without repair. Demographic, operative, and clinical data were collected through a review of medical records and patient surveys. All patients undergoing primary RSA with a minimum 2-year follow-up with a preoperative diagnosis of rotator cuff tear arthropathy, primary glenohumeral arthritis with cuff insufficiency (determined on preoperative exam, preoperative imaging, or intraoperatively), inflammatory arthritis, or massive rotator cuff tear were included. Patients were excluded for acute proximal humerus fractures, post-traumatic arthritis, and chronic dislocations. Patients were included regardless of the status of the subscapularis at the time of surgery, and, when present, the subscapularis was attempted to be repaired using the described technique in all cases.

Demographic and clinical data were collected through a review of medical records. These data included presence of comorbidities, status of rotator cuff evaluated intraoperatively, and postoperative complications. Active ROM was assessed preoperatively and postoperatively; ER, FE, and abduction were measured in degrees using a goniometer. IR was measured by determination of vertebral level and scored as described by Flurin et al^16^: no IR, 0; hip, 1; buttocks, 2; sacrum, 3; L4 to L5, 4; L1 to L3, 5; T8 to T12, 6; and T7 or higher, 7. Questionnaires for the pain visual analog scale, Quick Disabilities of Arm, Shoulder and Hand, American Shoulder and Elbow Surgeons score, Single Assessment Numeric Evaluation, and the Simple Shoulder Test were used to assess clinical function scores postoperatively. Postoperative data reported were collected at the date of the latest follow-up. Preoperative outcome scores were not included in this study due to a lack of preoperative outcome scores in a majority of patients.

### Radiographic analysis

Plain film x-rays and available advanced imaging (computed tomography and magnetic resonance imaging) were reviewed by a single evaluator (J.J.K.). Glenoid morphology was categorized according to the Walch classification using either two-dimensional or three-dimensional computed tomography reconstructions and was confirmed intraoperatively.^38^ Rotator cuff status was classified by the senior operating surgeon as intact, tendinosis, partial tearing, or full-thickness tearing based on preoperative imaging and/or intraoperative assessment.

### Surgical technique

The deltopectoral approach was utilized in all operations. Two different implants were used in the study—Exactech Equinoxe (Exactech, Gainesville, FL, USA) using a lateral humeral and medial glenoid design and the Integra Titan system (Smith + Nephew, Jacksonville, FL, USA) with a medial humeral and medial glenoid design. Both systems have a 145-degree neck-shaft angle. A subscapularis peel was performed for all operations with an intact subscapularis.

**Figure 1:**
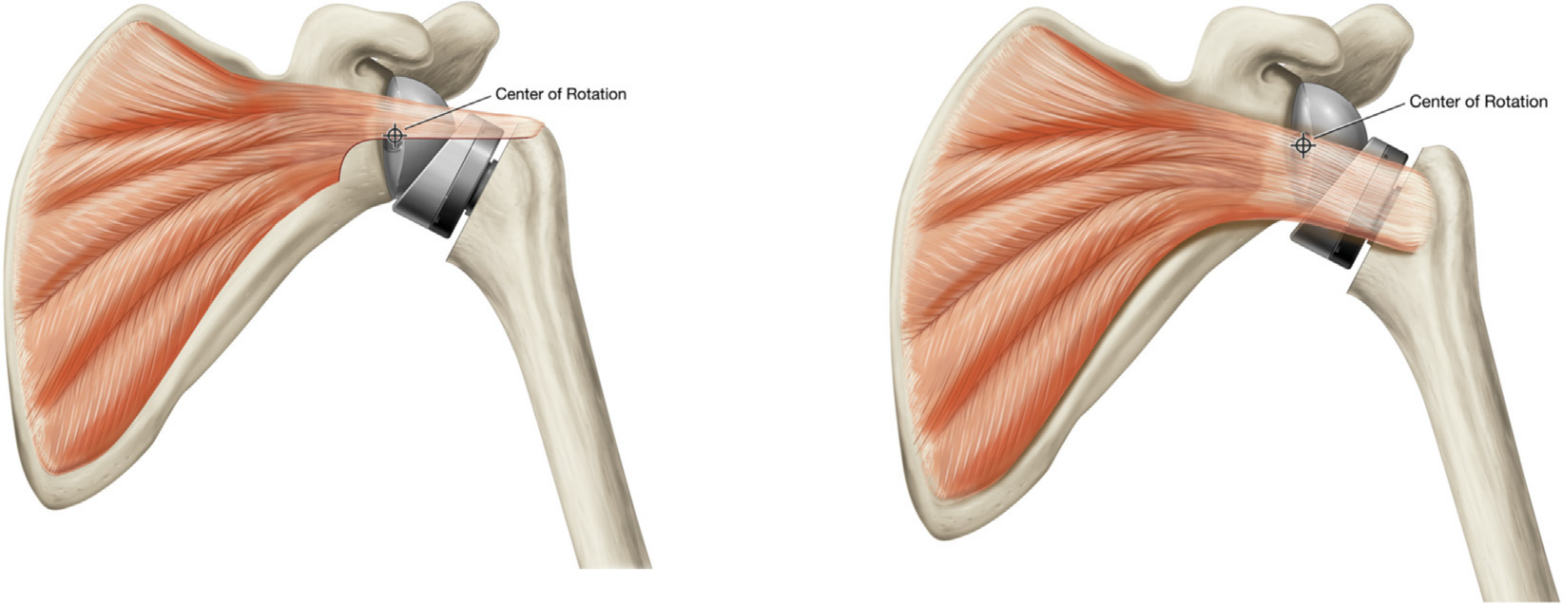
The over-the-top repair method of subscapularis repair (*left*) compared to the standard method of repairing the subscapularis (*right*).

The OTTR was performed as previously described by King et al.^21^ Following implantation and glenohumeral joint reduction, blunt dissection along the posterior, superior, and anterior borders of the subscapularis was performed just medial to the coracoid base to assure the coracohumeral ligament and any rotator interval scarring were released; the inferior border was not dissected to avoid injury to the axillary nerve. Using electrocautery, the inferior one-third was excised to allow for increased excursion and improved biomechanical abduction advantage.^21^ The remaining two-thirds of the tendon was then repaired to the over-the-top position defined as the anterior supraspinatus tendon or tendon remnant on the anterior greater tuberosity with interrupted nonabsorbable suture (Fig. 1). If no supraspinatus tissue was available for attachment, the subscapularis was attached to the anterior-most aspect of the greater tuberosity, just lateral to the bicipital groove using transosseous sutures. Repair was attempted in all patients; however, if the subscapularis was absent or torn intraoperatively or if a repair could not be performed with the shoulder in approximately 30 degrees of abduction and 20 degrees of ER without undue tension, then the subscapularis was not repaired.

### Rehabilitation

All patients completed an at-home postoperative exercise program as directed by a physical therapist. Pendulum exercises and passive forward flexion were started at 2 weeks postoperatively. Patients were advised to wear a sling for 6 weeks. ER was limited to neutral in all patients and IR past the abdomen was avoided for 6 weeks postoperatively. At 6 weeks postoperatively, ROM restrictions were lifted, active ROM movements were introduced, and the sling was discontinued. Strengthening was started at 12 weeks postoperatively.

### Statistics

Continuous measures were compared using Welch’s two-sided unpaired *t*-test and the Kruskal-Wallis rank-sum test, where appropriate. Count variables were compared using the Fisher’s exact test. The Holm method was used to correct for pairwise comparisons. Additionally, we determined the proportion of patients that had improvement at 2-year follow-up exceeding the minimal clinically important difference (MCID) and substantial clinical benefit (SCB) for ROM. Reference values were adopted from prior studies that utilized anchor-based methods.^35^^,^^36^ All statistical analysis were performed in R Core Team (version 4.2.0, R Foundation for Statistical Computing, Vienna, Austria). Significance was set at *P* < .05.

## Results

Ninety-seven patients were included in the analysis with 75 undergoing RSA with the OTTR and 22 treated without subscapularis repair (Table I), with a 77% rate of being able to perform the OTTR. Between the OTTR and no repair groups, respectively, there were no significant differences in age at surgery (69.4 ± 6.2 vs. 69.8 ± 5.2, *P =* .730), time to follow-up (3.8 ± 1.6 vs. 3.6 ± 1.7 years, *P =* .565), or sex (19% [N = 14] female vs. 5% [N = 1] female, *P =* .178). The preoperative diagnosis differed between patients who underwent OTTR vs. those who had no repair (*P* = .001); however, no significant differences were found when corrected for pairwise comparison. Significant differences were present between the preoperative status of superior and posterior rotator cuff muscles between the two groups, with full-thickness tears being more common in the no repair group. Furthermore, glenoid morphology was not significantly different between groups. Surgical factors of augment use, bone graft use, and blood loss were similar between the two groups (Table II).

**Table I:** Comparison of demographic, shoulder, and surgical characteristics between patients that underwent RSA with OTTR vs. those without repair of the subscapularis.

Characteristic	OTTR (n = 75)		No repair (n = 22)		*P* value
	N∗	Mean ± SD or % (N)	N∗	Mean ± SD or % (N)	
Age at surgery (y)	75	69.4 ± 6.2	22	69.8 ± 5.2	.730
Follow-up (mo)	75	3.8 ± 1.6	22	3.6 ± 1.7	.565
Sex, female	75	19% (14)	22	5% (1)	.178
Comorbidities					
Hypertension	75	72% (54)	22	68% (15)	.791
Heart disease	75	36% (27)	22	18% (4)	.129
Diabetes	75	32% (24)	22	36% (8)	.798
History of shoulder surgery	75	21% (16)	22	23% (5)	1.000
Preoperative diagnosis	75		22		**.001**
DJD		67% (50)		36% (8)	**.047**
Cuff-tear arthropathy		20% (15)		50% (11)	.071
MRCT		12% (9)		0% (0)	**.047**
Inflammatory arthritis		1% (1)		14% (3)	.114
Supraspinatus status	75		22		**.009**
Absent or full-thickness tear		27% (20)		55% (12)	.061
Partial tear		20% (15)		32% (7)	.384
Tendinosis		43% (32)		14% (3)	.051
Intact		11% (8)		0% (0)	.384
Infraspinatus status	75		22		.054
Absent or full-thickness tear		24% (18)		45% (10)	-
Partial tear		5% (4)		14% (3)	-
Tendinosis		11% (8)		9% (2)	-
Intact		60% (45)		32% (7)	-
Teres minor status	75		22		**.002**
Absent or full-thickness tear		5% (4)		27% (6)	**.016**
Partial tear		0% (0)		5% (1)	.227
Tendinosis		0% (0)		0% (0)	*NA*
Intact		95% (71)		68% (15)	**.007**
Walch classification	52		16		.207
A1		17% (9)		44% (7)	-
A2		13% (7)		6% (1)	-
B1		21% (11)		13% (2)	-
B2		31% (16)		13% (2)	-
B3		13% (7)		25% (4)	-
D		4% (2)		0% (0)	-

*DJD*, degenerative joint disease; *MRCT*, massive rotator cuff tear; *OTTR*, over-the-top subscapularis repair; *RSA*, reverse shoulder arthroplasty; *SD*, standard deviation.

Bold indicates statistical significance.

∗ N is given as the number of patients with available data.

**Table II:** Comparison of surgical characteristics between patients that underwent RSA with OTTR vs. those without repair of the subscapularis.

Characteristic	OTTR (n = 76)		No repair (n = 22)		*P* value
	N∗	Mean ± SD or % (N)	N∗	Mean ± SD or % (N)	
Augment used	75	55% (41)	22	64% (14)	.625
Glenoid bone graft	75	10.7% (8)	22	14% (3)	.708
Estimated blood loss (mL)	75	300 (200-400)	22	300 (250-388)	.843

*OTTR*, over-the-top repair; *RSA*, reverse shoulder arthroplasty; *SD*, standard deviation.

∗ N is given as the number of patients with available data.

### Range of motion and outcome scores

Patients that underwent OTTR showed improvement in all preoperative to postoperative ROM and outcome scores recorded (Table III). When assessing if outcomes for those patients reached the MCID, 82% of patients achieved the MCID for all ROM measures (Table IV). In addition, 67% achieved the SCB for all ROM measures.

**Table III:** Comparison of clinical outcomes between patients that underwent RSA with OTTR vs. those without repair of the subscapularis.

Outcome measure	OTTR (n = 75)		No repair (n = 22)		*P* value
	N∗	Mean ± SD	N∗	Mean ± SD	
Preoperative					
Active FE (°)	75	96 ± 33	21	90 ± 21	.376
Active abduction (°)	66	77 ± 29	18	77 ± 19	.983
Active ER (°)	73	15.3 ± 15.7	21	5.3 ± 11.7	**.003**
Active IR score	68	2 ± 2	20	2 ± 2	.317
Postoperative					
Active FE (°)	75	132 ± 21	22	126 ± 22	.268
Active abduction (°)	70	114 ± 26	21	106 ± 23	.193
Active ER (°)	69	28.1 ± 17.4	22	28.9 ± 20.0	.871
Active IR score	61	3 ± 2	19	2 ± 2	.089
SANE/SSV score	45	78.1 ± 20.2	10	77.4 ± 25.5	.940
QuickDASH score	39	32.9 ± 22.7	14	24.6 ± 21.3	.233
VAS score	58	2.3 ± 2.3	15	2.2 ± 2.5	.935
ASES score	57	72.9 ± 20.0	14	75.8 ± 22.0	.656
SST score	63	7.4 ± 3.4	16	7.1 ± 3.0	.729
Improvement					
Active FE (°)	75	36 ± 35	21	38 ± 28	.855
Active abduction (°)	61	36 ± 33	17	31 ± 33	.603
Active ER (°)	68	12.8 ± 20.3	21	24.2 ± 19.6	**.028**
Active IR score	54	1 ± 2	17	0 ± 2	.820

*ASES*, American Shoulder and Elbow Surgeons; *ER*, external rotation; *FE*, forward elevation; *IR*, internal rotation; *OTTR*, over-the-top subscapularis repair; *QuickDASH*, Quick Disabilities of the Arm, Shoulder and Hand; *RSA*, reverse shoulder arthroplasty; *SANE*, Single Assessment Numeric Evaluation; *SD*, standard deviation; *SST*, Simple Shoulder Test; SSV, subjective shoulder value; *VAS*, visual analog scale.

Bold indicates statistical significance.

∗ N is given as the number of patients with available data.

**Table IV:** Proportion of patients that exceeded the MCID and SCB for active ROM.

Outcome measure	Ref.†	OTTR (n = 75)		No repair (n = 22)		*P* value†
		N∗	% (N)	N∗	% (N)	
MCID						
Abduction (°)	−1.9	61	89% (54)	17	82% (14)	.682
FE (°)	−2.9	75	89% (67)	21	86% (18)	.701
ER (°)	−5.3	68	82% (56)	21	100% (21)	**.001**
SCB						
Abduction (°)	19.6	61	75% (46)	17	71% (12)	.756
FE (°)	22.3	75	67% (50)	21	71% (15)	.795
ER (°)	3.6	68	74% (50)	21	86% (18)	.379

*ER*, external rotation; *FE*, forward elevation; *MCID*, minimal clinically important difference; *OTTR*, over-the-top subscapularis repair; *SCB*, substantial clinical benefit; *ROM*, range of motion.

Bold indicates statistical significance.

∗ N is given as the number of patients with available data.

† Reference values adopted from Simovitch et al.

Preoperatively, patients undergoing OTTR had greater ER (15.3 ± 15.7° vs. 5.3 ± 11.7°, *P* = .003) compared to the no repair group (Table III). Postoperatively, patients undergoing OTTR had no difference in final ROM measures or final outcome scores. When comparing preoperative to postoperative improvement, however, patients in the OTTR group had greater improvement in ER (24.2 ± 19.6° vs. 12.8 ± 20.3°, *P* = .028), and patients with no subscapularis repair achieved the MCID more frequently for ER (100% (N = 21) vs. 82% (N = 56), *P* = .001) (Table IV), but achieved the SCB at a similar rate.

### Functional activities

Postoperative ability to perform activities of daily living involving IR and overhead motion was similar in patients who underwent OTTR and no repair (Table V). The single exception was a greater proportion of patients who underwent OTTR were able to reach the small of their back compared to those without repair (65% vs. 21%, *P* = .006). In patients that underwent OTTR, a majority reported either no, mild, or moderate difficulty in washing their back (55%, N = 28). A majority of OTTR patients found no difficulty when combing hair (72%, N = 39), managing toileting (59%, N = 32), and reaching a high shelf (56%, N = 30). Additionally, a majority also found washing their opposite axilla (61%, N = 33) and throwing overhand (52%, N = 28) to be either not difficult or somewhat difficult. Most patients were also able to place their hand behind their head with their elbow to the side (80%, N = 45), wash their opposite shoulder (52%, N = 29), and lift one pound to the level of their shoulder without bending their elbow (80%, N = 45). However, a majority were unable to throw overhand without the action affecting their shoulder, similar to the no repair group.

**Table V:** Postoperative comparison of activities involving internal rotation between patients that underwent RSA with OTTR vs. those without repair of the subscapularis.

Outcome measure	OTTR (n = 75)		No repair (n = 22)		*P* value
	N∗	% (N)	N∗	% (N)	
QuickDash					
Wash your back	51		19		.152
No difficulty		10% (5)		16% (3)	-
Mild difficulty		31% (16)		11% (2)	-
Moderate difficulty		14% (7)		21% (4)	-
Severe difficulty		35% (18)		16% (3)	-
Unable		10% (5)		37% (7)	-
ASES					
Wash your opposite axilla	54		14		.652
Not difficult		33% (18)		29% (4)	-
Somewhat difficult		28% (15)		43% (6)	-
Very difficult		19% (10)		7% (1)	-
Unable to do		20% (11)		21% (3)	-
Comb your hair	54		14		.764
Not difficult		72% (39)		86% (12)	-
Somewhat difficult		17% (9)		5% (1)	-
Very difficult		6% (3)		5% (1)	-
Unable to do		6% (3)		0% (0)	-
Manage toileting	54		14		.822
Not difficult		59% (32)		57% (8)	-
Somewhat difficult		35% (19)		36% (5)	-
Very difficult		4% (2)		7% (1)	-
Unable to do		2% (1)		0% (0)	-
Reach a high shelf	54		14		.217
Not difficult		56% (30)		50% (7)	-
Somewhat difficult		19% (10)		43% (6)	-
Very difficult		13% (7)		7% (1)	-
Unable to do		13% (7)		0% (0)	-
Overhand throw	54		13		.237
Not difficult		26% (14)		15% (2)	-
Somewhat difficult		26% (14)		54% (7)	-
Very difficult		13% (7)		0% (0)	-
Unable to do		35% (19)		31% (4)	-
SST					
Reach the small of your back	57		14		**.006**
Yes		65% (37)		21% (3)	-
Hand behind your head with the elbow to the side	56		14		.480
Yes		80% (45)		71% (10)	-
Lift one pound to the level of shoulder without bending your elbow	56		14		1.000
Yes		80% (45)		86% (12)	-
Overhand throw without affecting the extremity	56		14		.321
Yes		32% (18)		14% (2)	-
Wash back of opposite shoulder	56		14		.766
Yes		52% (29)		43% (6)	-

Bold indicates statistical significance.

*QuickDASH*, Quick Disabilities of the Arm, Shoulder and Hand; *ASES*, American Shoulder and Elbow Surgeons; *SST*, Simple Shoulder Test; *RSA*, reverse shoulder arthroplasty; *OTTR*, over-the-top subscapularis repair.

∗ N is given as the number of patients with available data.

### Complications

Complications and reoperations did not significantly differ between patients undergoing OTTR vs. no repair (12.0% [n = 9] vs. 13.6% [n = 3], *P* = 1 and 4.9% [n = 3] vs. 4.5% [n = 1], *P* = 1). Complications in patients undergoing OTTR included coracoid bursitis, humeral loosening requiring revision to endoprosthesis, two cases of instability, periprosthetic fracture, three cases of unexplained pain, and one case of ulnar nerve paresthesias. Complications in the group with no repair included instability without dislocation, one case of unexplained pain, and one case of recurrent subluxations.

## Discussion

Whether the chosen management of the subscapularis during RSA has a clinically meaningful influence on patient outcomes remains a point of contention among shoulder surgeons. A biomechanical study we performed investigating this topic determined that OTTR of the subscapularis imparts an improved abduction moment through a wide ROM compared to anatomic repair, so in this study, we aimed to assess clinical outcomes in patients that underwent RSA with concomitant OTTR of the subscapularis and secondarily compare them to patients that did not receive repair of the subscapularis. Patients who underwent OTTR demonstrated improvement in all ROM measures and functional outcome scores. Additionally, a majority of these patients achieved the MCID and SCB for all ROM measures. Compared to the no repair group, final ROM and outcome scores were similar between groups; however, the no repair group gained significantly more ER while the OTTR group showed improved functional IR, with a significantly greater proportion reporting being able to reach the small of their back.

Patients undergoing OTTR showed similar postoperative outcomes compared to ranges reported in the literature after RSA. This was evidenced by comparable functional outcome scores between OTTR vs. previously reported Single Assessment Numeric Evaluation (78.1 ± 20.2 vs. 75.4-85^14^^,^^20^^,^^23^^,^^24^), Quick Disabilities of Arm, Shoulder and Hand (32.9 ± 22.7 vs. 21-36.1^3^^,^^9^^,^^24^^,^^33^), visual analog scale (2.3 ± 2.3 vs. 0.4-2.6^3^^,^^9^^,^^17^^,^^20^^,^^25^^,^^28^), American Shoulder and Elbow Surgeons (72.9 ± 20.0 vs. 62-83^9^^,^^20^^,^^25^^,^^28^^,^^30^^,^^32^), and Simple Shoulder Test (7.4 ± 3.4 vs. 6-9.1^14^^,^^20^^,^^24^) scores. Similar findings were also present for ROM, including FE (132 ± 21° vs. 110-153°^20^^,^^25^^,^^28^^,^^32^^,^^33^), abduction (114 ± 26° vs. 98-111°^9^^,^^33^^,^^34^), and ER (28.1 ± 17.4° vs. 22-52°^9^^,^^14^^,^^20^^,^^25^^,^^28^). These findings further support the use of OTTR as a viable option to manage the subscapularis while maintaining the known effectiveness of RSA.

Studies investigating the biomechanics of an anatomic subscapularis repair have shown that reattachment leads to deltoid antagonism and a greater adduction moment arm during abduction in all three RSA designs evaluated.^1^^,^^18^ Thus, to mitigate the unfavorable biomechanics of a anatomic subscapularis repair, biomechanical studies have investigated insertion at a more superior location to alter the subscapularis moment arm.^13^^,^^21^ In our previous study,^21^ we found that when the upper two-thirds of the subscapularis was repaired to the anterior aspect of the greater tuberosity, it imparted a greater biomechanical abduction advantage when compared to native placement. Similarly, Eno et al^13^ determined biomechanical abduction and FE advantage when moving the subscapularis to a superior location on the lesser tuberosity. The current study supports these biomechanical studies by showing that superiorization of the subscapularis through OTTR of the subscapularis procedure leads to similar postoperative FE and abduction compared to no repair, suggesting no significant antagonistic effect. Additionally, we also found a slight functional improvement in IR with OTTR compared to no repair, with an increased ability to reach the small of the back (Table V). Unfortunately, our series did not include patients undergoing standard repair thus comparison of patients with a standard repair to patients with OTTR was not possible; future studies are needed to assess whether OTTR may be advantageous over a standard repair when indicated and when possible.

Tenotomy of the subscapularis without repair led to a greater improvement of ER when compared to OTTR. This was not unexpected, as repair of the subscapularis can limit ER, particularly in lateralized designs or patients with a noncompliant subscapularis tendon.^4^^,^^26^^,^^37^ This relationship can likely be attributed to the increased ER force needed to overcome the added resistance of the repaired subscapularis (ie, force couple). Additionally, while lateralized RSA designs (as compared to Grammont-style designs) increase the tension on the anterior and posterior deltoid heads,^31^ possibly allowing the deltoid to impart the necessary torque for axial rotation, medialized RSA may be more reliant on the rotator cuff to accomplish axial rotation.^5^ Thus, it is possible that OTTR may be advantageous over no repair when a medialized (Grammont-style) RSA design is used. Physicians might tailor their decision to repair the subscapularis to individual circumstances and patient needs, such as in patients that have diminished ER and/or higher ER demands.

Though supporters of subscapularis repair theorize that it reduces risks of dislocation due to increased anterior stability, the current study did not show this. Prior studies have shown that this may be implant design-dependent, particularly being important for globally medialized designs.^11^^,^^15^^,^^22^^,^^39^ Implants that lateralize the center of rotation increase compressive forces of the deltoid which theoretically provides increased global stability to reduce the chance of dislocation in addition to tensioning any remaining rotator cuff; since the most common implant used in this study is a medialized glenoid with a lateralized humerus design, the improved stability imparted by subscapularis repair may not have been functionally meaningful.^15^^,^^29^ However, for globally medialized designs, OTTR may impart the benefit of improved stability without the detrimental addition of the larger antagonistic adduction moment created by anatomic repair.

We understand that there are limitations to this study, the most notable being its retrospective nature and limited sample size. Furthermore, management of the patients’ subscapularis was not randomized, and cohorts were not able to be matched by preoperative diagnosis or rotator cuff status. Additionally, many of the patients in the tenotomy without repair group had a functionally irreparable subscapularis, which may be associated with other conditions that have an influence on clinical outcomes. Therefore, selection bias may have been introduced when patients were able to receive OTTR, and the generalizability of the findings may be somewhat limited. Additionally, our proportion of male participants is far greater (85%) than the average population of RSA patients (36%). This difference owes to the patient population being from the Veterans Health Administration Hospital System, where 95% of the patients are males.^2^ In addition, a lack of preoperative outcome scores precluded evaluation of the improvement in outcome scores among the groups which further limits this study. Furthermore, two different implants were utilized in the study as well as different repair techniques depending on the available soft tissue on the anterior aspect of the great tuberosity. While these factors improve generalizability, differences in implant design and repair techniques may have confounded the influence of OTTR on postoperative functional outcomes. Additionally, due to the retrospective nature of this study, we did not prospectively evaluate subscapularis integrity postoperatively, and, therefore, failure of the OTTR may have confounded our results. However, precautions were taken to aid healing of the repaired subscapularis, including patients remaining in a sling with ROM limitations for the first 6 weeks postoperatively.

## Conclusion

Management of the subscapularis during RSA remains controversial. RSA with the over-the-top subscapularis repair produced reliable improvements in ROM with good pain relief and functional outcome scores, though we found only minor advantage over no repair, with slightly improved functional IR but with less improvement in ER. This study shows the feasibility of this novel subscapularis repair without a detriment to patient outcomes, but further studies with longer follow-up and more patients are needed to see in which situations it may impart additional clinical benefit compared to anatomic subscapularis repair or no repair.

## Disclaimers:

Funding: No sources of funding were disclosed by the authors.

Conflicts of interest: Dr. Schoch has a consultancy agreement with Exactech, Inc., and receives royalties from Exactech, Inc., Innomed and Responsive Arthroscopy. Dr. King is a paid consultant for Exactech, Inc. and LinkBio Corp. Mr. Hao has a consultancy agreement with LinkBio Corp. The other authors, their immediate families, and any research foundation with which they are affiliated have not received any financial payments or other benefits from any commercial entity related to the subject of this article.
